# TGF-β regulation of the uPA/uPAR axis modulates mesothelial-mesenchymal transition (MesoMT)

**DOI:** 10.1038/s41598-021-99520-5

**Published:** 2021-10-27

**Authors:** Ranisha Logan, Ann Jeffers, Wenyi Qin, Shuzi Owens, Prashant Chauhan, Satoshi Komatsu, Mitsuo Ikebe, Steven Idell, Torry A. Tucker

**Affiliations:** Department of Cellular and Molecular Biology, The University of Texas Health Science Center at Tyler, 11937 US HWY 271, Biomedical Research Building, Lab A-5, Tyler, TX 75708 USA

**Keywords:** Imaging, Cell division, RNAi, Transcription, Biological techniques, Cell biology, Molecular biology, Physiology, Pathogenesis

## Abstract

Pleural fibrosis (PF) is a chronic and progressive lung disease which affects approximately 30,000 people per year in the United States. Injury and sustained inflammation of the pleural space can result in PF, restricting lung expansion and impairing oxygen exchange. During the progression of pleural injury, normal pleural mesothelial cells (PMCs) undergo a transition, termed mesothelial mesenchymal transition (MesoMT). While multiple components of the fibrinolytic pathway have been investigated in pleural remodeling and PF, the role of the urokinase type plasminogen activator receptor (uPAR) is unknown. We found that uPAR is robustly expressed by pleural mesothelial cells in PF. Downregulation of uPAR by siRNA blocked TGF-β mediated MesoMT. TGF-β was also found to significantly induce uPA expression in PMCs undergoing MesoMT. Like uPAR, uPA downregulation blocked TGF-β mediated MesoMT. Further, uPAR is critical for uPA mediated MesoMT. LRP1 downregulation likewise blunted TGF-β mediated MesoMT. These findings are consistent with in vivo analyses, which showed that uPAR knockout mice were protected from *S. pneumoniae*-mediated decrements in lung function and restriction. Histological assessments of pleural fibrosis including pleural thickening and α-SMA expression were likewise reduced in uPAR knockout mice compared to WT mice. These studies strongly support the concept that uPAR targeting strategies could be beneficial for the treatment of PF.

## Introduction

Pleural fibrosis (PF) is a chronic and progressive lung disease, likely as a result of inflammation of the pleural space subsequent to injury or infection^1,2^. PF, if advanced, results in lung restriction, dyspnea, and can ultimately cause respiratory compromise. In these injury states, normal pleural mesothelial cells (PMCs) generally undergo a phenotypic change referred to as mesothelial mesenchymal transition (MesoMT). Through this transition, the relatively quiescent mesothelial cell acquires a profibrotic phenotype and begin expressing the myofibroblast markers; α-smooth muscle actin (SMA), collagen and fibronectin^3–8^. These mesothelial cell-derived myofibroblast, in conjunction with florid fibrin deposition, play a critical role in organization and remodeling within the pleural cavity^3–8^.

The urokinase-type plasminogen activator receptor (uPAR) is a 55-60 kDa glycoprotein that is expressed at the cellular surface of diverse cell types, including pleural mesothelial cells^9,10^. It is composed of 3 extracellular domains (D1, D2, and D3), which can be catalytically cleaved, between domains 1 and 2, by its ligand, the urokinase-type plasminogen activator (uPA) and plasmin. uPA is a serine protease that activates plasminogen to its active form, plasmin, through proteolysis. Binding to uPAR is reported to increase the enzymatic activity of uPA^11^. uPAR cleavage by uPA exposes a sequence reported to increase cellular migration^12–15^. The binding of uPA to uPAR is also known to induce migration, adhesion, and proliferation through the association of transmembrane proteins, such as the G protein-coupled formyl peptide receptor 1, integrins and the epidermal growth factor receptor (EGFR)^13–16^. Further, we reported that enhanced uPAR expression contributed to increased tumor virulence in our orthotopic model of malignant pleural mesothelioma^10^.

Previous studies in our laboratory have demonstrated that extracellular uPA reduces uPAR protein half-life in HPMCs^12^. Specifically, uPA reduces uPAR half-life by greater than 50%. Further, uPA-mediated uPAR decreases in protein half-life were mediated by interactions with the low-density lipoprotein receptor–related protein 1 (LRP1). LRP1 is a 600kDA surface receptor, that can bind and internalize a wide array of surface ligands including uPA, Tissue-type plasminogen activator (tPA) and the uPA/uPAR/PAI-1 complex. Although proinflammatory mediators like tumor necrosis factor alpha (TNF-α) and interleukin (IL)-1β can induce uPAR expression in mesothelial and mesothelioma cells through transcriptional regulation, we found that these mediators also reduce LRP1 expression^12^. Consequently, uPAR protein half-life was increased in the presence of TNF-α and IL-1β. LRP1 can also be neutralized by binding to receptor associated protein (RAP), an intracellular LRP1 chaperone. We showed that RAP treatment likewise increased uPAR half-life in pleural mesothelial cells (PMCs), thus prolonging uPA enzymatic activity (1). However, the role of uPA/uPAR/LRP1 in the progression of MesoMT has not been investigated.

In this study, the roles of uPAR and uPA were investigated by downregulating their respective expression using targeted small interfering RNA (siRNA) transfection. Gene knockdown was confirmed using immunoblot analysis and quantitative real-time polymerase chain reaction (qPCR). The enzyme-linked immunosorbent assay (ELISA) was used to measures changes in uPA expression. We found that uPAR and uPA are critical to the progression of MesoMT. How these components regulate the progression of MesoMT remains unclear and to our knowledge has not been previously studied in the context of pleural remodeling.

## Methods

### PMC culture conditions and treatment

Permission to collect and use human (H)PMCs was granted through an exempt protocol approved by the Institutional Human Subjects Review Board of the University of Texas Health Science Center at Tyler. All experiments were performed in accordance with relevant guidelines and regulations. HPMCs were isolated from pleural fluids collected from patients with congestive heart failure or that were post-coronary bypass pleural effusions, as previously described^17^. These cells were maintained in LHC-8 culture media (Life Technologies, Carlsbad CA) containing 3% fetal bovine serum (Life Technologies), 2% antibiotic–antimycotic (Life Technologies) and GlutaMAX (Life Technologies), as previously reported^4,5,12,17–19^. Mesothelial cell purity was determined by measuring the expression of calretinin (> 95%), prior to use in experiments. Transfections were performed as previously described^3–5,20^. Briefly, uPAR and uPA were downregulated using Lipofectamine 3000 with control or the respective siRNA (Table 1, 200 nM). Cells then recovered in standard culture media. Cells were then serum starved prior to use in experiments.

**Table 1:** Includes the sequence and reference information for the select siRNAs used in the knockdown studies.

Gene	siRNA Sequence
*PLAUR*	GCCGUUACCUCGAAUGCAUUU AUGCAUUCGAGGUAACGGCUU
*PLAU*	GAGAUCACUGGCUUUGGAA UUCCAAAGCCAGUGAUCUC
*LRP1*	CAUCGAUCUUCACAAAGGA UCCUUUGUGAAGAUCGAUG
Negative control (Sigma)	Mission siRNA negative control #2

### *Streptococcus pneumoniae*-mediated model of pleural injury

All experiments involving animals were approved by the Institutional Animal Care and Use Committee at the University of Texas Health Science Center at Tyler. All experiments were performed in accordance with relevant guidelines, policies, and regulations. Pleural injury was initiated by *S. pneumonaie* infection, as previously described^6^. Wild-type C57BL/6 (WT) mice and uPAR knockout mice (Plaur^tm1Jld^, 10–12 weeks of age, ≈20 g, Jackson Laboratory, Bar Harbor ME) were lightly anesthetized with isoflurane. Intrapleural inoculations (1.8 × 10^8^ cfu, suspended in 0.9% saline) of *Streptococcus pneumoniae* (*S. pneumoniae*, D39, National Collection of Type Cultures, Salisbury UK) were delivered by intrapleural injection in 150 µL saline, as previously described^5,6,20,21^. Amoxicillin was administered daily, for 4 days after infection. Lung function and volumes were collected at the conclusion of the experimental course at 7d, as previously reported^3,5,6,22^.

### Lung histology and imaging

All de-identified human pleural tissues were obtained from the National Disease Research Interchange from surgical biopsy or autopsy specimens from patients with a clinical diagnosis of nonspecific pleuritis or from patients with histologically near-normal pleural tissues that were resected for reasons unrelated to pleural disease or from individuals who died from causes unrelated to any pleural pathologic process. Lung histology and immunostaining were performed as previously described^6,18,23^. Briefly, all tissue sections (human and murine) were first deparaffinized and subjected to antigen retrieval using a citrate buffer at 95 °C for 20 min. Tissue analyses, collagen deposition and localization were initially assessed by Trichrome staining, as previously described^18,23^.

Morphometric analyses of pleural tissue thickness were performed on Trichrome stained tissues, as we previously described^18^. Immunofluorescence was used to visualize uPAR (AM1, a generous gift from Andrew Mazar) expression in human normal and pleuritis pleuropulmonary sections^5,18^. Similar studies were performed on lung tissue sections from our mouse model of pleural fibrosis (Goat anti mouse polyclonal AF534, R&D). Fluorescence images were obtained using a Leica TSC SP8 confocal laser scanning microscopy system (Leica Microsystems, Inc., Heidelberg, Germany). A series of optical sections were collected at 1 μm intervals in Z-axis (17 μm). These multiple Z-series sections were then projected onto one plane at 25 × optical zoom, as previously described^4,12,18,20^.

For surface imaging of uPAR, fluorescence-activated cell sorting (FACS) was used as previously described^12^. Briefly, untransfected, control siRNA and uPAR siRNA transfected HPMCs were labeled with a rabbit IgG or rabbit anti human uPAR polyclonal antibody (AM1). Cells were then labeled with an Alexa 647-labeled donkey α-rabbit secondary antibody. Cells were then sorted using a Millipore Easycyte HT flow cytometer and data graphed using FlowJo.

### Western blotting

Serum-starved HPMCs were treated with TGF-β (5 ng/ml, R&D Minneapolis MN), thrombin (7 nM, Enzyme Research Laboratory, South Bend, IN), Factor Xa (Xa, 7 nM, Enzyme Research Laboratory), urokinase plasminogen activator (uPA, 20 nM, Sekisui Lexington MA) or plasmin (7 nM, Molecular innovations). Cell lysates were then western blotted for α-SMA (MAB1420, R&D), uPAR (AM1), and phosphorylated Akt-Serine 473 (4060, Cell Signaling), as previously described^18,24^. Total Akt (4061, Cell Signaling) and β-actin (A1978, Sigma-Aldrich) was used as the loading control.

### qPCR analyses

Total RNA was isolated from treated HPMCS using the Qiagen RNeasy kit and transcribed into cDNA, as previously described^6,24^. uPAR (*PLAUR*), uPA (*PLAU*), LRP1 and α-SMA (*ACTA2*) gene expression was then determined by qPCR analyses on a Bio-Rad CFX Touch (Table 2). GUSB and GAPDH were used as loading controls.

**Table 2:** Includes the reference information for the genes used in the detailed qPCR analyses.

Primer	Sequence
uPA (*PLAUR*)	Bio-Rad Prime PCR Probe Assay: Cy5 Fluorophore, qHsaCIP0031103
uPA (*PLAU*)	Bio-Rad Prime PCR Probe Assay: Cy5.5 Fluorophore, qHsaCIP0032518
*LRP1*	ACATATAGCCTCCATCCTAATC GCTTATACCAGAATACCACTC
α-SMA (*ACTA2*)	Bio-Rad Prime PCR Probe Assay: FAM Fluorophore, qHsaCIP0028813
GUSB	Bio-Rad Prime PCR Probe Assay: HEX Fluorophore, qHsaCIP0028142

### Enzyme-linked immunosorbent assay (ELISA)

A human uPA total antigen ELISA kit (HUPAKT-TOT, Molecular Innovations) was used measure changes in uPA expression by HPMCs. Protein lysates were quantified via BCA to normalize the protein load. Cell lysates (12 µg of protein) and the uPA standard curve provided were added to antibody coated microplates. The ELISA was then performed following manufacturer’s instructions. Absorbance was measured at 450 nm and uPA protein concentrations were determined using the standard curve according to manufacturer’s instructions.

### Cleavage resistant uPAR construct

A cleavage resistant mutant of uPAR was used to determine the role of uPAR cleavage on the induction of MesoMT. The cleavage resistant (CR) uPAR construct was designed by mutating four known protease cleavage sites: R91K, Y87C, R89K, and R83K, as reported by Mazzieri et al.^14^, with some modifications, using VectorBuilder. The adenovirus (AdV) was next amplified and purified using the PureVirus™ Adenovirus Purification Kit, according to manufacturer’s instructions (Cell Biolab). The purified virus was then quantified, aliquoted and stored at − 80 °C. HPMCs word 2 MOI of GFP-AdV, WT-uPAR-AdV (WT) and CR-uPAR. Cells were then serum-starved and treated with TGF-β for 24 h (RNA) or 48 h (protein).

### Statistics

All statistics were performed using the ANOVA, Student t-test or Mann Whitney U test. A p-value of less than 0.05 was considered significant. This study was reported in accordance with ARRIVE guidelines.

## Results

### uPAR expression is robust in human pleuritis

**Figure 1:**
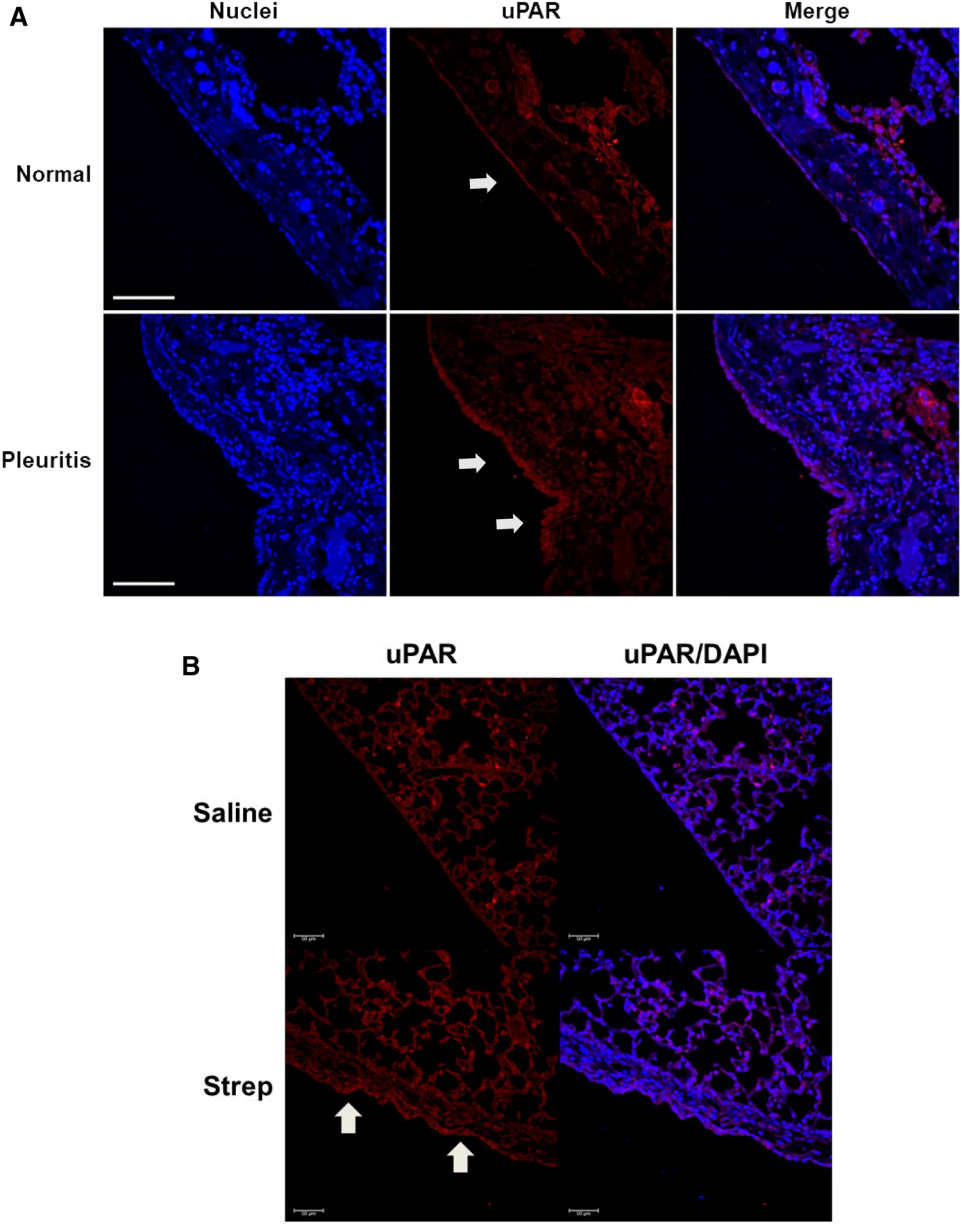
uPAR is expressed in normal and human nonspecific pleuritis. Lung tissue sections from normal patients and patients diagnosed with nonspecific pleuritis were immunofluorescently labeled for uPAR. (**A**) uPAR expression (cyan) was detected at the pleural surface of nonspecific pleuritis tissue and normal pleural tissues. Images are × 25 and are representative of the findings of 30 fields/slide and 3–4 patients/group. Solid arrows indicate areas of uPAR positivity along the pleural surface. White bar indicates 100 µm. (**B**) uPAR expression (red) was detected in lung sections of *S. pneumonaie* infected and saline treated WT mice. Images are at × 25 and are representative of the findings of 15 fields/slide and 3–4 mice/treatment. Solid arrows indicate areas of uPAR positivity along the pleural surface. White bar indicates 50 µm.

We have previously reported that pleural reorganization in both human pleuritis and our mouse models of pleural fibrosis is characterized by the appearance of myofibroblasts and the accumulation of extracellular matrix proteins such as collagen 1^3–6,24–26^. We further showed that pleural mesothelial cells play an active role in disease progression. However, the role of uPAR in these processes has not been investigated. We first measured uPAR expression at the pleural surface of normal and pleuritis human lung tissues. (Fig. 1A). We found that uPAR expression was robust in both normal and pleuritis mesothelial lining tissues. Further this expression was limited to the pleural surface with focal areas of positivity throughout the lung. In our mouse models of pleural injury (Fig. 1B), we found that uPAR expression was not detectable at the surface of the normal unperturbed pleural mesothelium. Conversely, uPAR expression was robust at the pleural surface in *S. pneumonaie* infected mice by 7 days. These studies suggest that uPAR expression is enhanced in pleural injury.

### Characterization of uPAR expression in HPMCs

**Figure 2:**
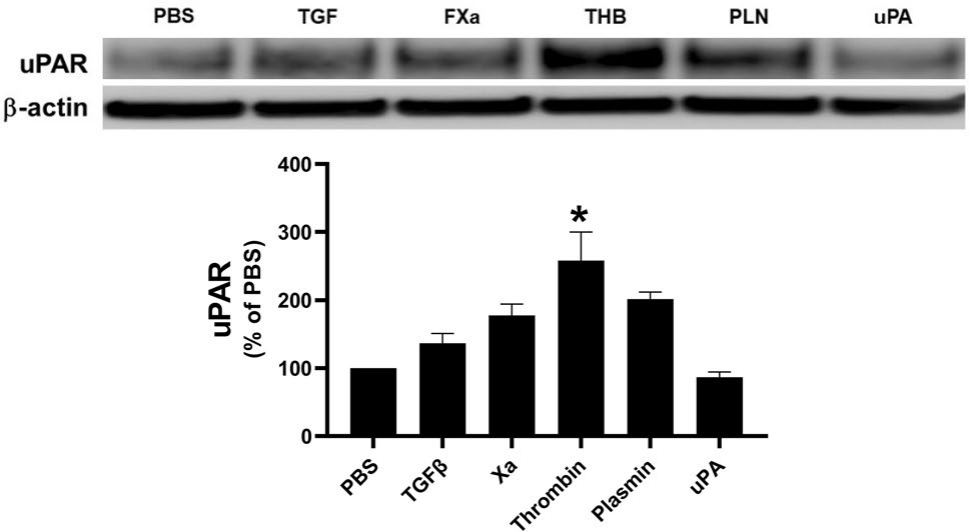
uPAR expression is modulated by mediators of MesoMT. Serum-starved HPMCs were treated with PBS, TGF-β (5 ng/ml), FXa (Xa, 13 nM), thrombin (THB, 7 nM), plasmin (PLN, 7 nM), and uPA (20 nM) for 48 h. uPAR expression was then determined by western blotting. β-actin was used as the loading control. TGF-β, FXa, thrombin and plasmin treated cells demonstrated increased uPAR expression. Image is representative of three independent experiments. Graphed densitometric analysis represents n = 3.

Because uPAR expression was detected in the pleural mesothelium, we next measured uPAR expression in primary HPMCs ex vivo. We first characterized the expression of uPAR in cells treated with mediators previously shown to induce MesoMT: TGF-β, Factor Xa, thrombin, plasmin and uPA^18^. While uPAR expression was detectable in PBS treated cells, the relative expression was low. Of the selected mediators, thrombin alone significantly induced uPAR expression in primary HPMCs tested (Fig. 2).

### uPAR knockdown blocks MesoMT

**Figure 3:**
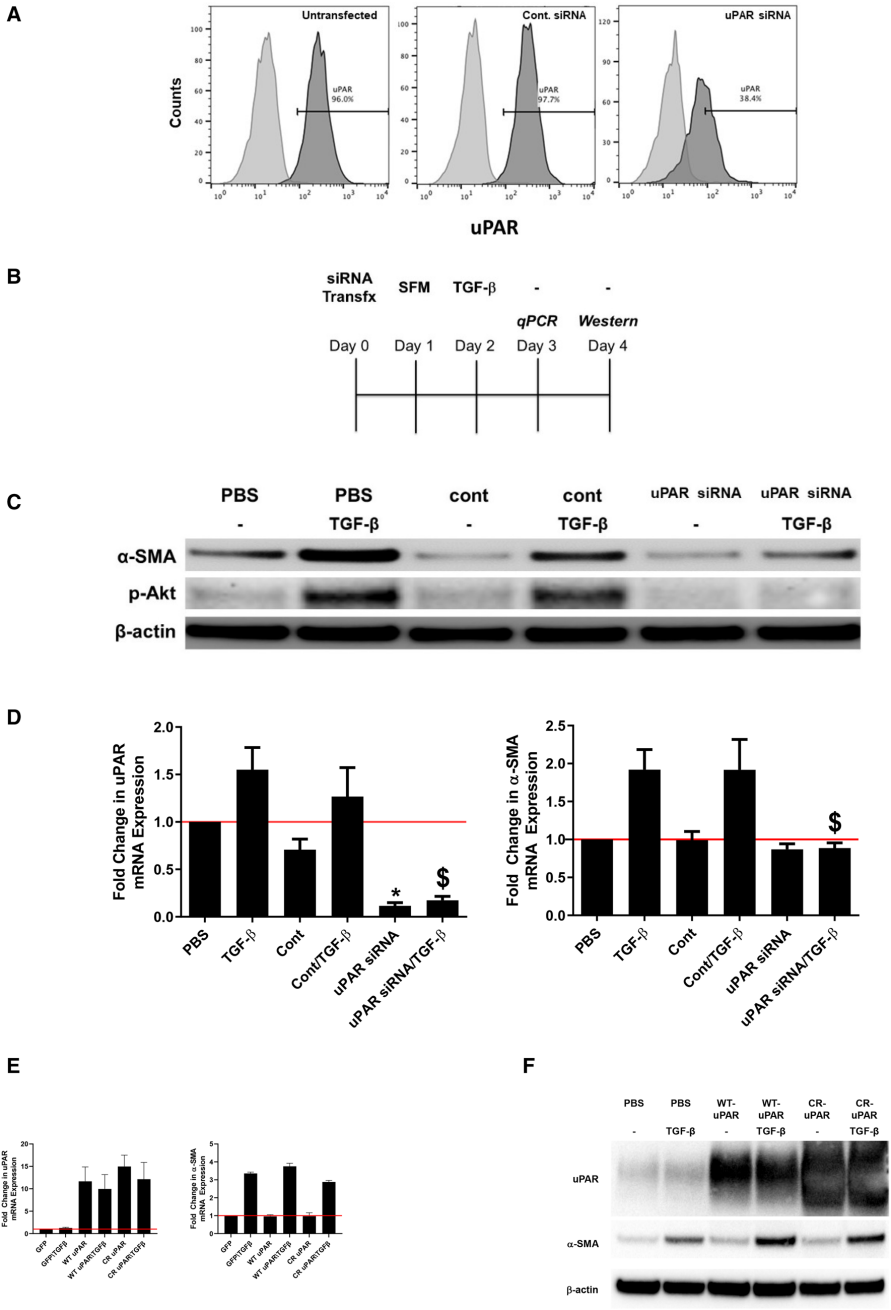
uPAR down-regulation blocks TGF-β induced MesoMT. Untransfected, control siRNA and uPAR siRNA transfected cells were serum-starved for 24 h. (**A**) Cells were then labeled for surface uPAR via FACs. (**B**) Diagram of cell transfection and treatment schedule. Serum-starved untransfected, control siRNA and uPAR siRNA transfected cells were treated with PBS or TGF-β for 24 h (RNA analyses) and 48 h (protein analyses). (**C**) Cell lysates were resolved by SDS-PAGE and immunoblotted for α-SMA and phosphorylated Akt via Western blot. Akt was the loading control. Image is representative of three independent experiments. (**D**) Total RNA was isolated from untransfected, control siRNA and uPAR siRNA transfected cells treated with TGF-β for 24 h. Changes in α-SMA and uPAR mRNA levels were determined by qPCR analyses. GUSB was used as the reference gene. Data represents the mean of three independent experiments. *denotes p < 0.05 compared to PBS control. $ denotes p < 0.05 compared to TGF-β treated cells. (**E**) HPMCs were treated with GFP-, WT-uPAR, or cleavage resistant uPAR adenovirus. Serum-starved cells were then treated with TGF-β for 24 h. Changes in α-SMA and uPAR mRNA levels were determined by qPCR analyses. GUSB was used as the reference gene. Data represents the mean of three independent experiments. (**F**) HPMCs were treated with GFP, WT-uPAR, or cleavage resistant uPAR adenovirus. Serum-starved cells were then treated with TGF-β for 48 h. Changes in α-SMA and uPAR were determined by Western blot. β-actin was the loading control.

After determining that uPAR expression was robust in human pleuritis and our *S. pneumoniae* mouse model of PF, we next determined the role of uPAR in MesoMT. We first confirmed that siRNA targeting could reliably reduce uPAR expression. Using FACs, we found that uPAR expression was dramatically reduced by uPAR siRNA targeting (Fig. 3A). After confirming uPAR knockdown, untransfected, control siRNA and uPAR siRNA transfected cells were treated with TGF-β (Fig. 3B). Immunoblot analyses showed that TGF-β induced α-SMA in untransfected and control siRNA treated cells (Fig. 3C). Conversely, similarly treated uPAR siRNA cells demonstrated only a modest change in α-SMA expression. We previously reported that the PI3K/Akt pathway was critical for induction of MesoMT^24^. As such, we assayed the lysates for changes in Akt phosphorylation in uPAR downregulated HPMCs. TGF-β induced Akt phosphorylation in control HPMCs. Conversely, TGF-β mediated Akt phosphorylation was muted in uPAR down-regulated HPMCs. These findings were confirmed by parallel qPCR analyses that show uPAR downregulation (p < 0.05) blocked TGF-β induced α-SMA (Fig. 3D).

We next evaluated the effect of uPAR overexpression in the induction of MesoMT. Because uPAR cleavage by uPA has been reported to mediate some cellular responses, we also investigated the role of the uPAR cleavage in the induction of MesoMT. Using adenoviral transduction, uPAR expression was enhanced by tenfold (Fig. 3E). After confirming enhanced uPAR expression, GFP, WT-uPAR and cleavage resistant (CR) uPAR expressing cells were treated with TGF-β. TGF-β mediated increases in α-SMA were not affected by enhanced uPAR expression. Immunoblot analyses confirmed this finding, showing that uPAR expressing cells demonstrated similar TGF-β mediated changes in α-SMA (Fig. 3F).

### uPA Knockdown blocks induction of MesoMT

**Figure 4:**
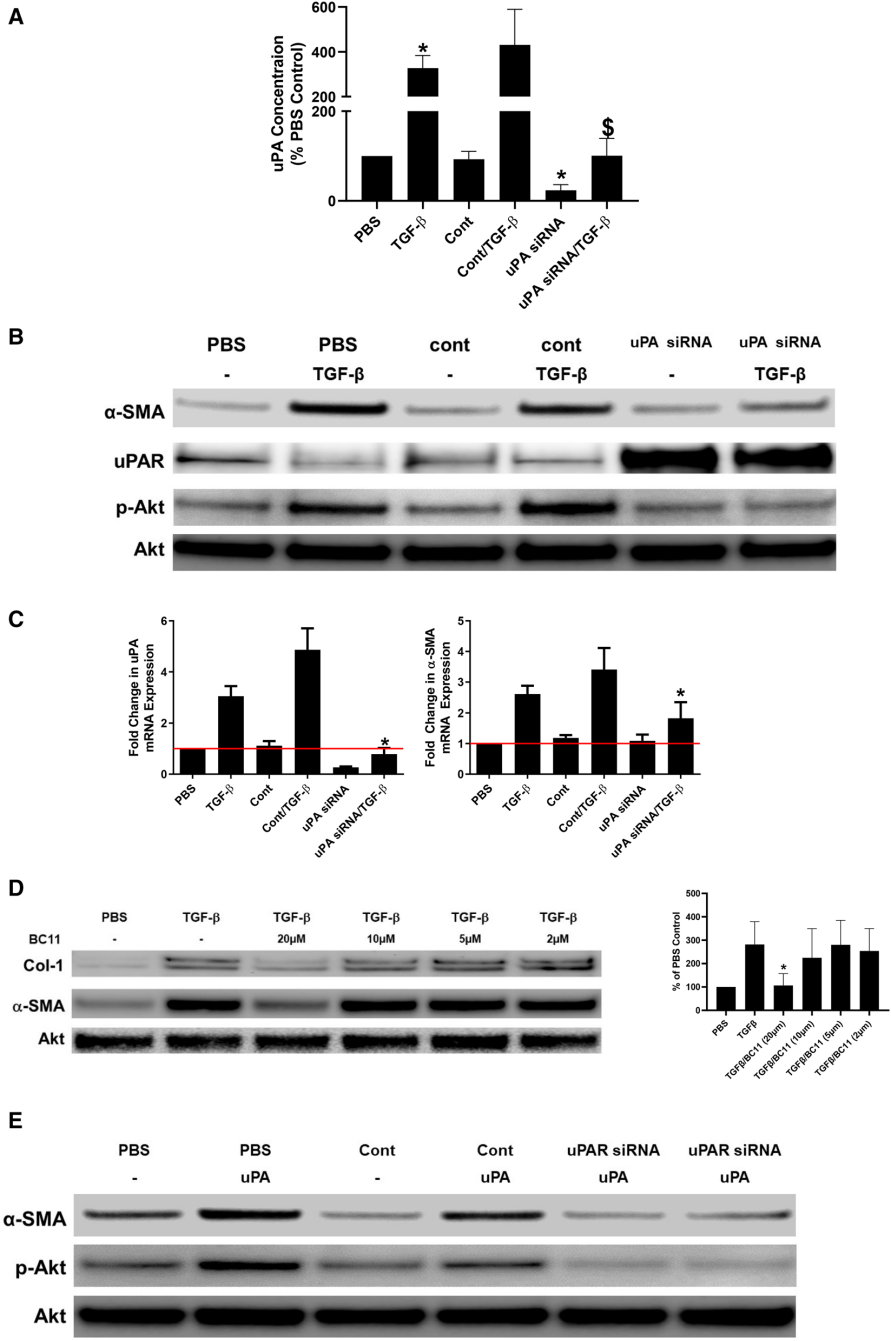
uPA down-regulation blocks TGF-β induced MesoMT. Untransfected, control siRNA and uPA siRNA transfected cells were serum-starved for 24 h. (**A**) Cell lysates were then probed for urokinase antigen by ELISA. Serum starved untransfected, control siRNA and uPA siRNA transfected cells were treated with PBS or TGF-β. Serum starved untransfected, control siRNA and uPA siRNA transfected cells were treated with PBS or TGF-β for 24 h (RNA analyses) and 48 h (protein analyses). (**B**) Cell lysates were resolved by SDS-PAGE and immunoblotted for α-SMA, uPAR and phosphorylated Akt via Western blot. Akt was the loading control. Image is representative of three independent experiments. (**C**) Total RNA was isolated from untransfected, control siRNA and uPA siRNA transfected cells that had been treated with TGF-β for 24 h. Changes in α-SMA and uPA mRNA levels were determined by qPCR analyses. GUSB was used as the reference gene. Data represents the mean of three independent experiments. *denotes p < 0.05 compared to TGF-β treated HPMCs. HPMCs were serum-starved in the presence or absence of the uPA inhibitor, BC11, for 24 h. Cells were then treated with TGF-β in the presence and absence of BC11 for 48 h. (**D**) Cell lysates and conditioned medias were resolved by SDS page and immunoblotted for α-SMA and Col-1, respectively, via Western blot. β-actin was the loading control. Image is representative of three independent experiments. Graphed data represent n = 3. * denotes a p < 0.05 compared to TGFβ treatment. (**E**) Serum starved untransfected, control siRNA and uPAR siRNA transfected cells were treated with PBS or uPA for 48 h. Cell lysates were resolved by SDS-PAGE and immunoblotted for α-SMA and phosphorylated Akt via Western blot. Akt was the loading control. Image is representative of three independent experiments.

We previously reported that uPA, among other mediators, induces MesoMT^18^. Because knockdown of uPAR, blocked induction of MesoMT (Fig. 3), we next sought to determine the role of uPA in TGF-β mediated MesoMT and specifically whether it involved interaction with uPAR. For these studies, we first confirmed that uPA siRNA could reliably reduce uPA expression in TGF-β treated HPMCs. Untransfected, control siRNA and uPAR siRNA transfected cells were treated with TGF-β for 48 h. ELISA of these lysates showed that uPA expression was significantly increased by TGF-β treatment compared to PBS treated HPMCs (Fig. 4A, p < 0.05). Further, we found that uPA siRNA significantly reduced baseline and TGF-β induced uPA compared to untransfected HPMCs (p = 0.02 and p = 0.01, respectively). After confirming uPA knockdown, untransfected, control siRNA and uPA siRNA transfected cells were treated with TGF-β for 48 h and probed for markers of MesoMT (Fig. 4B). While TGF-β induced α-SMA expression in the control cells, uPA downregulated cells showed reduced α-SMA induction. TGF-β mediated induction of Akt phosphorylation was likewise reduced in uPA downregulated HPMCs. The findings were also confirmed by parallel qPCR analyses (Fig. 4C).

Because uPA knockdown blocked TGF-β-mediated induction of MesoMT, we next evaluated the ability of uPA inhibition to block the TGF-β response. In these experiments, HPMCs were treated with the uPA inhibitor, BC11, prior to treatment with TGF-β. While TGF-β induced α-SMA and Col-1, uPA inhibition diminished the response (Fig. 4D). Specifically, the highest dose of BC11 (20 µm) significantly reduced TGF-β mediated increases in α-SMA. Similar results were seen with TGF-β mediated increased in Col-1. Parallel qPCR analyses confirmed that uPA inhibition blocked TGF-β mediated MesoMT (data not shown). BC11 also reduced baseline αSMA and Col-1 protein expression in HPMCs (data not shown). These findings strongly suggest that uPA activity is likewise critical for the induction of TGF-β mediated MesoMT.

Because both uPAR and uPA knockdown attenuated TGF-β mediated MesoMT, we next considered the role of uPAR in uPA-mediated MesoMT. Untransfected, control, and uPAR siRNA transfected cells were treated with uPA. As previously reported, uPA treatment induced α-SMA expression in untransfected and control siRNA transfected cells (Fig. 4E). Conversely, uPAR downregulation blocked uPA associated increases in α-SMA. Further, uPA mediated induction of Akt phosphorylation was likewise blocked in uPAR downregulated HPMCs. These studies strongly suggest that the uPA/uPAR axis is an important interaction for the progression of MesoMT.

### LRP1 knockdown blocks TGF-β mediated MesoMT

**Figure 5:**
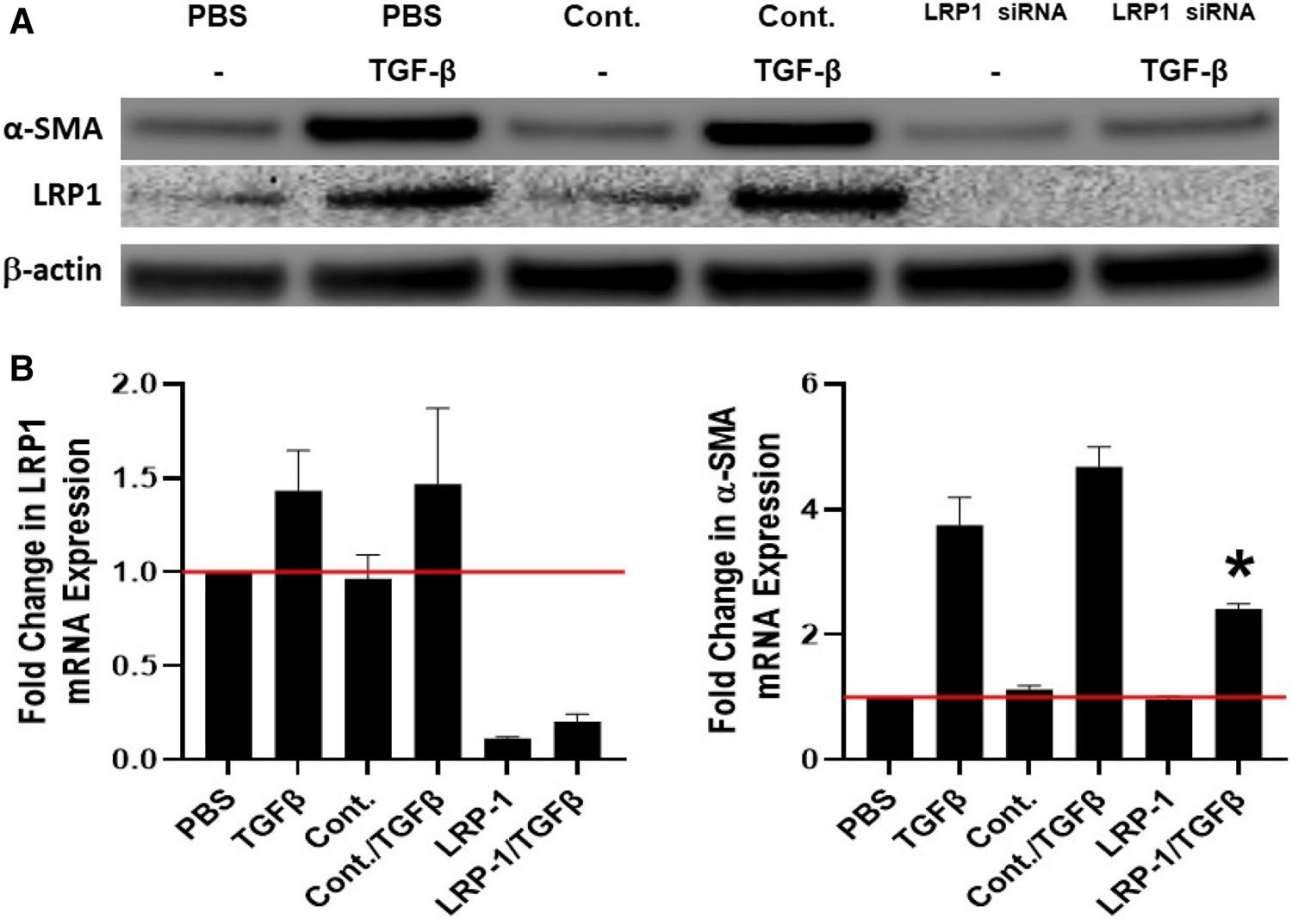
LRP1 down-regulation blocks TGF-β induced MesoMT. Untransfected, control siRNA and LRP1 siRNA transfected cells were serum-starved for 24 h. Serum-starved untransfected, control siRNA and LRP1 siRNA transfected cells were treated with PBS or TGF-β for 24 h (RNA analyses) and 48 h (protein analyses). (**A**) Cell lysates were resolved by SDS-PAGE and immunoblotted for α-SMA Western blot. Akt was the loading control. Image is representative of three independent experiments. (**B**) Total RNA was isolated from untransfected, control siRNA and LRP1 siRNA transfected cells treated with TGF-β for 24 h. Changes in α-SMA and LRP1 mRNA levels were determined by qPCR analyses. GUSB was used as the reference gene. Data represents the mean of three independent experiments. *denotes p < 0.05 compared to siRNA control.

Because uPAR and uPA are critical for the induction of MesoMT, we sought to further elucidate this mechanism in MesoMT. uPAR lacks a cytoplasmic domain and requires a surface binding partner for signal transduction. We previously reported that LRP1 regulates uPAR on the cell surface^12^. As such we next determined its role in TGF-β mediated MesoMT. Untransfected, control siRNA and LRP1 siRNA transfected HPMCs were treated with TGF-β (Fig. 5). Immunoblot analyses showed that LRP1 was downregulated by targeting siRNA. We also found TGF-β induced α-SMA in untransfected and control siRNA treated cells. Conversely, similarly treated LRP1 siRNA cells demonstrated only a modest change in α-SMA expression (Fig. 5A). Similar results were found in parallel qPCR analysis (Fig. 5B). These studies suggest that LRP1 may play a role in uPA/uPAR mediated MesoMT.

### uPAR deficiency protects against *S. pneumoniae* induced PF

**Figure 6:**
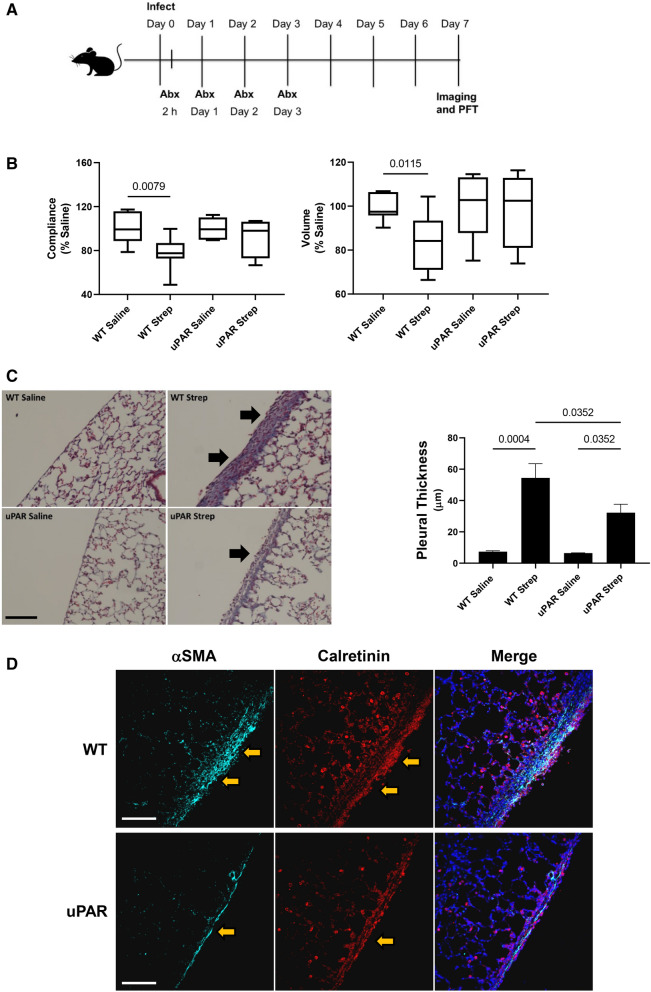
uPAR deficiency attenuates the progression of empyema-mediated pleural fibrosis. (**A**) Diagram of infection and treatment. WT and uPAR knockout mice were intrapleurally injected with 1.8 × 10^8^ cfu of *S. pneumoniae*. Treatment with antibiotics (Abx) was began 2 h after infection and daily for the next 3 days. After a 7d time course, lung volumes and function were evaluated by CT scan and Scireq flexivent, respectively. (**B**) uPAR deficiency blocked significant *S. pneumoniae* mediated decrements in lung volume and function. Tissue sections from *S. pneumoniae* injured WT and uPAR deficient mice were next trichrome stained to characterize changes in lung architecture and collagen deposition. (**C**) *S. pneumoniae*-mediated pleural injury induced significant pleural thickening in WT and uPAR deficient mice compared to saline treated control mice. uPAR knockout mice demonstrated significantly reduced pleural thickening compared to WT mice. N = 5–7 mice/group 15 fields/slide. Solid arrows indicate areas of pleural thickening. Bar indicates 100 µm. (**D**) Tissue sections from *S. pneumoniae* injured WT and uPAR deficient mice were stained for the MesoMT marker α-SMA (cyan) and the mesothelial cell marker, calretinin (red). uPAR knockout mice demonstrated reduced α-SMA expression compared to similarly treated WT mice. N = 5–7 mice/group 15 fields/slide. Bar indicates 100 µm. Solid arrows indicate areas of pleural thickening.

We previously reported that *S. pneumonaie* infection significantly reduced lung volume and lung function in our mouse model of pleural fibrosis^3–6^. Because uPAR knockdown attenuated TGF-β mediated MesoMT, we next evaluated the role of uPAR in this murine PF model (Fig. 6A). *S. pneumoniae* infected WT mice demonstrated significant decrements in lung function compared to saline treated controls (Fig. 6B, p < 0.05). Lung volumes were likewise significantly reduced by *S. pneumonaie* infection. Conversely, *S. pneumoniae* mediated changes in lung function and volume in uPAR deficient mice were not significant, indicative of a protective effect.

Histologic analyses of WT and uPAR KO lung tissues showed significant pleural thickening, resulting from *S. pneumoniae* infection, in both the WT and uPAR deficient mice (Fig. 6C). However, the pleural thickening observed in uPAR deficient mice was significantly less than that observed in infected WT mice. Further, tissue sections from WT mice demonstrated higher levels of the MesoMT marker α-SMA compared to uPAR knockout mice, which colocalized with calretinin expressing mesothelial cells (Fig. 6D). Discrete areas of calretinin positivity were found within the lung parenchyma away from the pleural surface. This staining likely represents macrophages, which have been reported to cross-react with calretinin antibodies^27^. These data show that uPAR deficiency protects against *S. pneumonaie*-mediated MesoMT and subsequent pleural remodeling.

## Discussion

We and others have previously shown that increased uPAR expression contributes to aggressive cellular phenotypes^10,12–16,28^. Further, increased uPAR expression has also been shown to be associated with increased tumor virulence both in vitro and in vivo. Other studies have shown that uPAR signaling can induce cancer stem cell-like properties in carcinoma cell lines, either simultaneously or independent of epithelial-mesenchymal transition (EMT)^10,16^. Cancer studies have shown that uPAR induces mesenchymal genes in cancer cells, such as glioblastoma^16,29,30^. Our prior work showed that uPAR expression correlated with increased tumor virulence in malignant pleural mesothelioma^10^. We have also shown that uPA promotes MesoMT in human and murine PMCs^18^. Further, studies using an uncleavable uPAR mutant showed that uPA dependent uPAR cleavage is critical for cell migration^14^. Although numerous studies have investigated the role of uPAR in aggressive cellular phenotypes, its contribution to the progression of MesoMT and PF remains unclear. Thus, representing a potentially important gap in knowledge in the field.

We first characterized the expression of uPAR in human pleuritis tissues. While variability due to disease severity, timing of sample collection, and the etiology of the pleuritis complicated these analyses, we found that uPAR expression was enhanced in diseased tissue sections. Further, pleural thickening was also observed in the pleuritis lung tissues compared to the normal. In mouse studies, uPAR was relatively absent from the pleural mesothelium in controls but enhanced after *S. pneumoniae* infection. This discrepancy may be due to the differences by which the tissues were collected. While mouse tissues were harvested immediately upon euthanization, most human tissues utilized for these studies were collected within 24–48 of death. As such, some aberrant staining due to decomposition may occur. Further, while the listed diagnosis maybe normal, other underlying disease(s) may be present. In some cases, “normal” tissues are isolated from donors without a diagnosis of pleural injury or infection; however, other underlying diseases/conditions that promote inflammation, such as cancer, may confound this “normal” characterization. As such, the saline treated mouse controls may more accurately represent true normalcy. Although changes in uPAR expression were modest in human tissues, regulation of its ligand, uPA, could likely modulate its activity, providing rationale for the study of both.

In our previous reports, we found numerous inflammatory mediators are rapidly upregulated in our *S. pneumonaie* model of pleural fibrosis. However, with antibiotic intervention these mediators peak at 3d and quickly subside to baseline level by day 7^6^. By the 7d timepoint the injury model is in its resolution/fibrotic phase, which is driven by TGF-β. In our published reports, downregulation of the endogenous uPA inhibitor, PAI-1, increased the severity of pleural fibrosis^6,18^. Specifically, PAI-1 deficiency in our carbon black/bleomycin model of pleural injury significantly increased pleural thickening and worsened decrements in lung function. Conversely, PAI-1 overexpression promoted fibrin deposition but modestly improved pleural thickening^18^. Similar results were observed in the *S. pneumoniae* PF model, as PAI-1 deficiency robustly induced inflammatory mediators and caused early death^6^. These results support our hypothesis that TGF-β mediated increases of uPA contributes to the progression of PF. Although, the effect of TGF-β on uPAR expression is modest, it is the most reliable mediator of MesoMT. Further, TGF-β uniquely induces uPA expression. For these reasons TGF-β was selected as the best mediator to evaluate the role of the uPAR/uPA axis in MesoMT.

To directly determine the role of uPAR in TGF-β mediated MesoMT, we next downregulated uPAR expression in HPMCs using siRNA targeting and confirmed expression via FACs analyses. uPAR knockdown was found to block induction of MesoMT in HPMCs by TGF-β. Because we previously reported that PI3K/Akt signaling was critical for the induction of MesoMT^24^, we next probed cell lysates for changes in PI3K/Akt signaling. As anticipated, the PI3K/Akt signaling pathway was likewise reduced in uPAR downregulated cells. These studies strongly suggest that uPAR expression is an important determinant in TGF-β mediated MesoMT.

Because the recognized ligand for uPAR is uPA, and uPA was significantly increased in TGF-β treated cells, we next evaluated its role in MesoMT. After confirming uPA knockdown by siRNA targeting, we evaluated the progression of TGF-β mediated MesoMT in uPA knockdown cells. Like uPAR, uPA knockdown blocked the induction of MesoMT. PI3K/Akt signaling was likewise blunted in uPA knockdown cells. Further, uPA knockdown increased uPAR expression. We previously reported that uPA reduced the half-life of uPAR and destabilized its expression^12^. These data suggest that baseline uPAR expression on HPMCs is likely reduced by endogenously secreted uPA. Similarly, reductions in uPAR observed in TGF-β treated cells are also likely due to TGF-β mediated increases in uPA, which then downregulate uPAR expression. While uPA was identified as an important mediator of TGF-β mediated MesoMT, it is an enzymatically active protein with diverse modes of action. BC11 is a small molecule inhibitor of uPA, which mimics the actions of PAI-1. In our studies, we found that BC11 blocked induction of MesoMT and reduced baseline αSMA and Col-1. These studies suggests that baseline and induced uPA contribute to the profibrotic phenotype observed in HPMCs. To further elucidate the role of uPA in MesoMT, we next determined the role of uPAR in uPA induced MesoMT. These studies showed that uPAR was a necessary player in uPA induced MesoMT, as uPA could not induce MesoMT in uPAR downregulated HPMCs. uPA is currently being used to treat pleural loculations due to complicated parapneumonic effusions or empyema. However, this enzymatic activity is not durable and is rapidly neutralized^31^, which would limit its contribution to pleural reorganization. Collectively these data show that TGF-β mediated induction of uPA is a critical determinant in the progression of MesoMT. Further, this effect is likely via uPA/uPAR interactions.

While uPAR is reported to contribute to diverse cellular functions^9,10,12,32^, it lacks a cytoplasmic domain that is required for intracellular signal transduction. As such, uPAR is reported to mediate signaling via interaction with other surface receptors, such as LRP1^33^. We previously reported that LRP1 neutralization increased uPAR protein stability in HPMCs^12^. uPA enzymatic activity was likewise modulated by LRP1 expression. Accordingly, we next interrogated the role of LRP1, a surface binding partner for uPAR, in MesoMT. Our results presented here show that LRP1 downregulation, like uPA and uPAR, blocked TGF-β mediated MesoMT. However, these blockade effects were less pronounced than those observed in uPA or uPAR knockdown HPMCs (Fig. 5B). While, the interactions between uPA and uPAR are direct and interdependent^10,12,16,29,32,33^, how uPAR mediates its signaling is less clear. Signaling via uPAR is reported to be mediated via interactions with multiple surface receptors, not LRP1 alone. EGFR is also reported to be an important mediator in the uPA/uPAR axis in diverse cell types^14,29^. While these studies suggest a role for LRPP1 in uPA/uPAR signaling, other surface receptors may also contribute to these effects.

With the abundance of in vitro data suggesting uPAR’s role in MesoMT, we next evaluated the role of uPAR in the progression of pleural fibrosis in our previously reported *S. pneumoniae* model of PF^3,5,6^. As expected, *S. pneumoniae*-mediated pleural injury significantly decreased lung function and reduced lung volumes in WT mice. These significant decrements were likely a consequence of increased pleural thickening resulting from *S. pneumonaie* mediated injury. The MesoMT marker α-SMA was likewise increased in the thickened pleura of WT mice compared to uPAR deficient mice. In contrast, these significant decrements in lung function were muted in uPAR knockout mice. Although pleural thickening was still apparent in uPAR deficient mice, it was significantly attenuated compared to WT mice. These findings suggest that uPAR contributes to the progression of pleural fibrosis and thickening, a prognosticator of poor outcomes in clinical injury. Further, this process likely involves interactions with uPA, and merit continued investigation.

## Supplementary Information

Supplementary Figures.

Figure Deck for the manuscript.
