# Supplementary figures and images for: TGF-β regulation of the uPA/uPAR axis modulates mesothelial-mesenchymal transition (MesoMT)
Source: Sci Rep. 2021 Oct 27;11:21210. doi: 10.1038/s41598-021-99520-5 (PMC8551303; doi:10.1038/s41598-021-99520-5)

# Figure 2

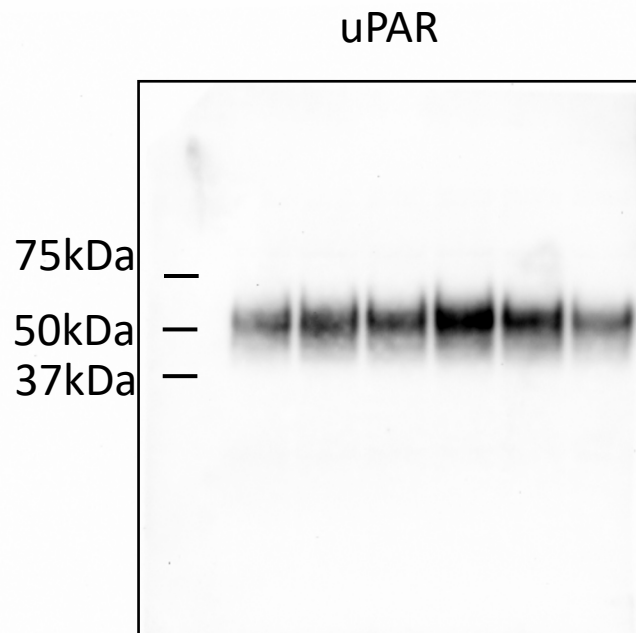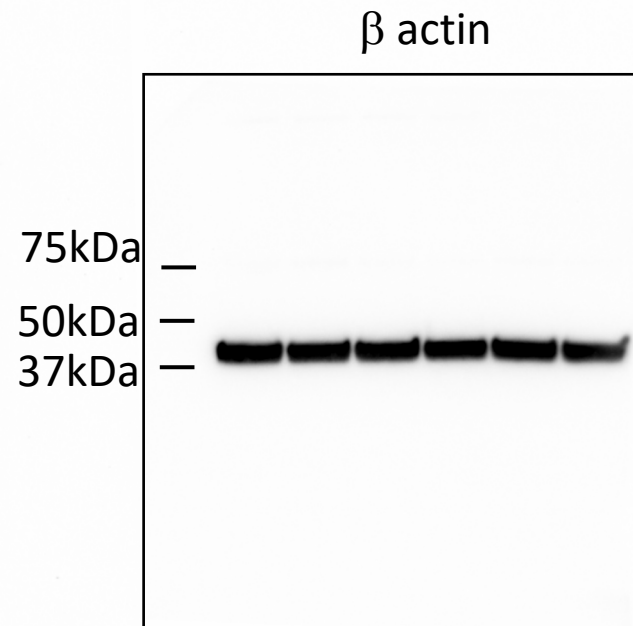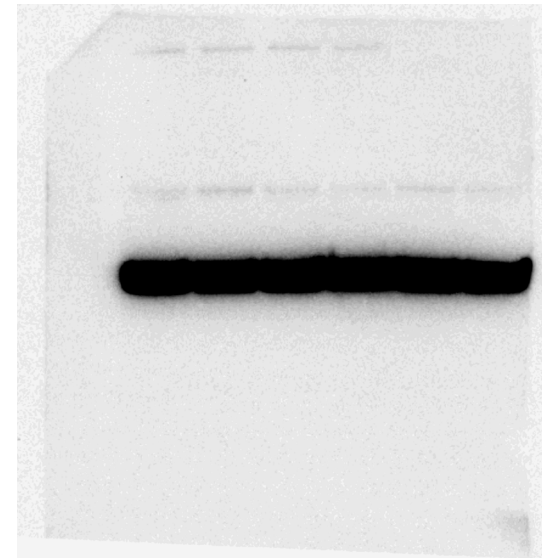

# Figure 3

p-AKT

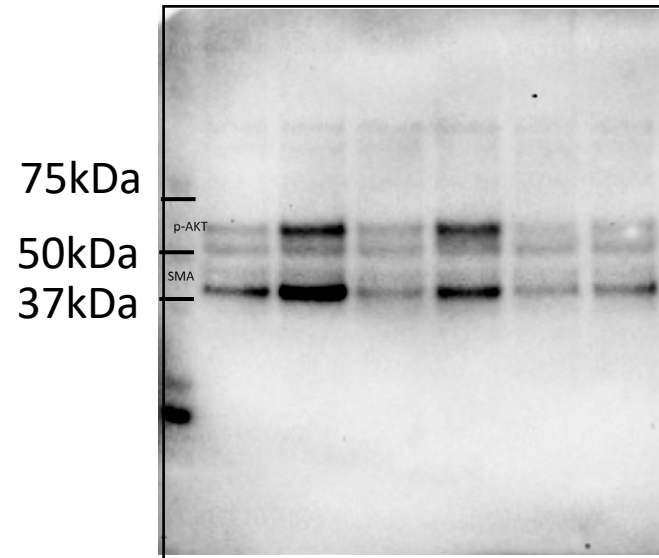

$\alpha$ -SMA

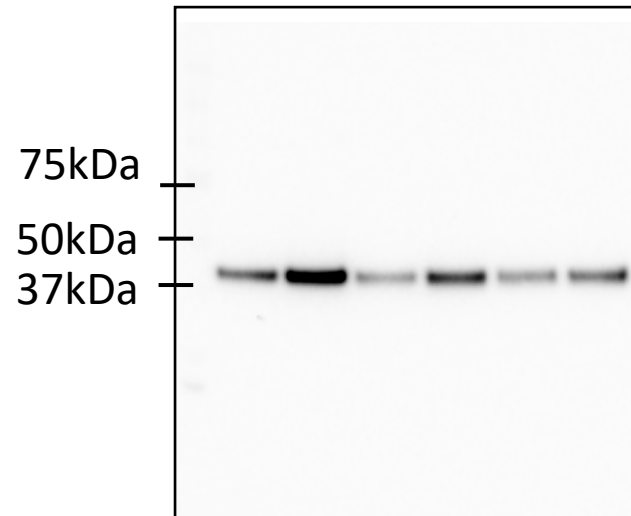

$\beta$ -actin

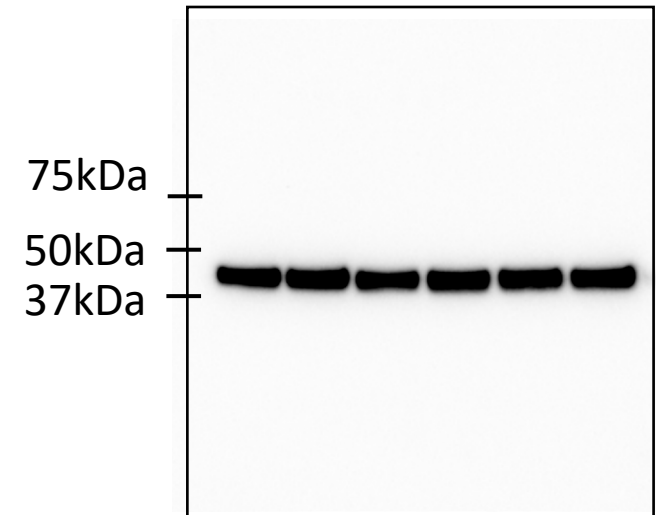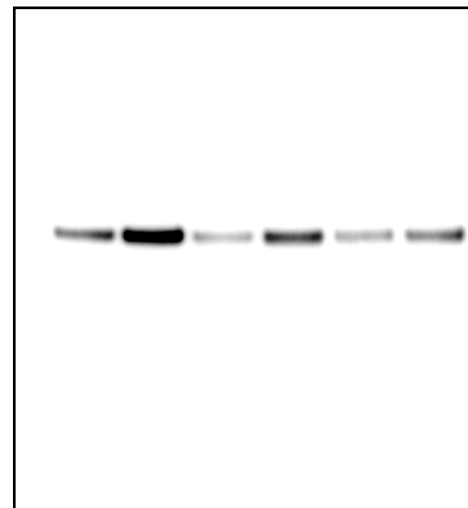

# Figure 3E Continued

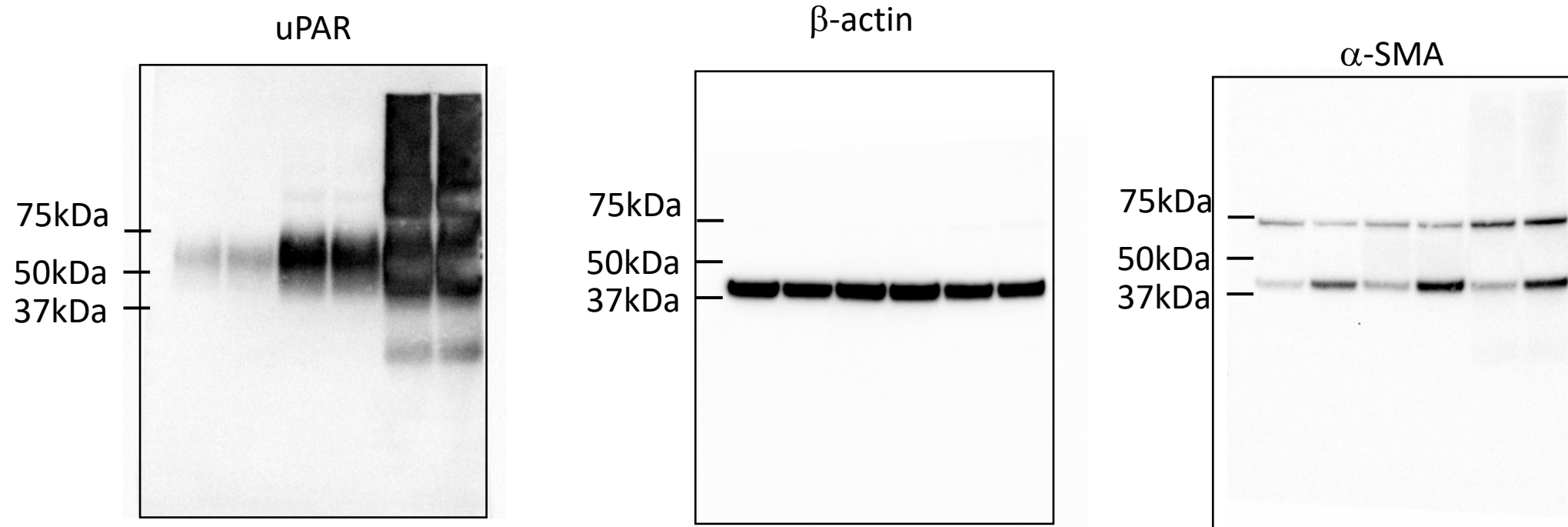

# Figure 4

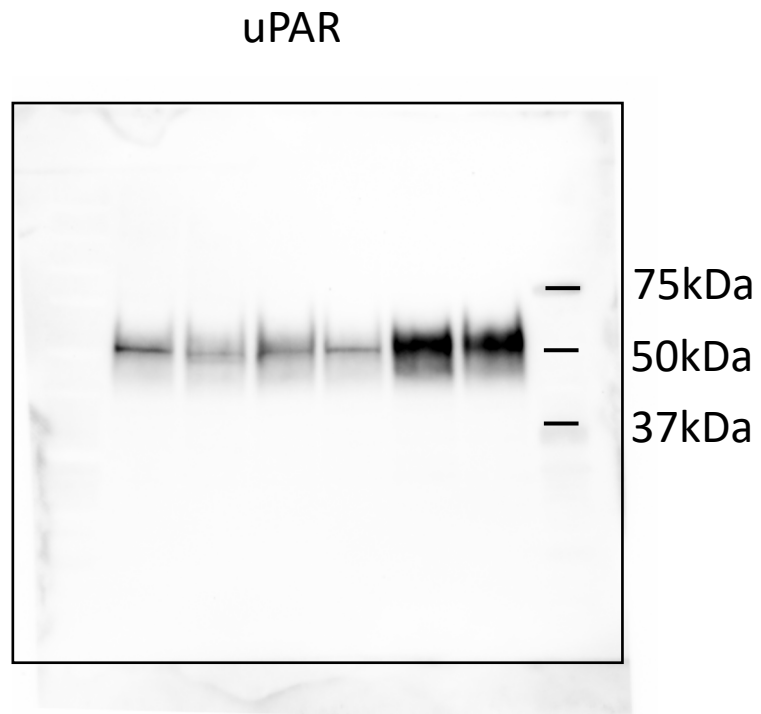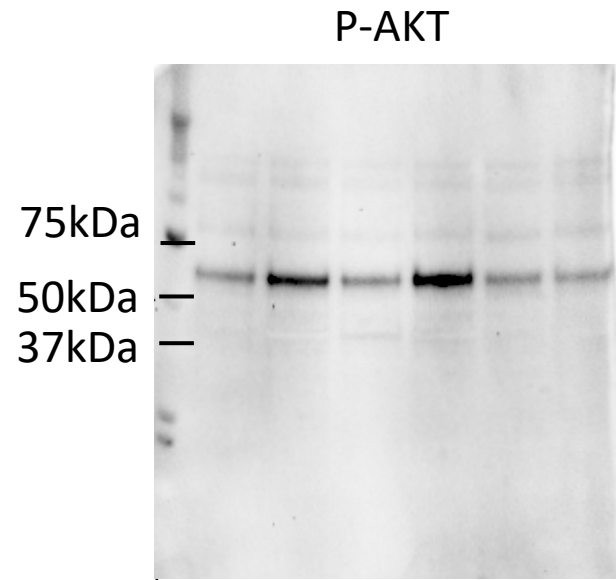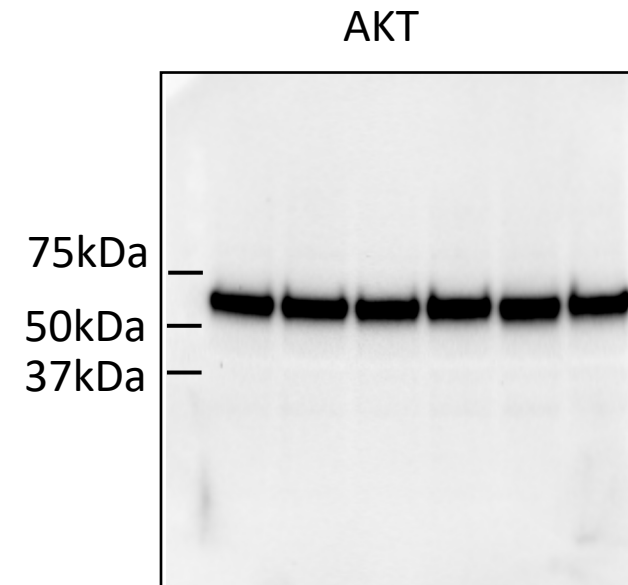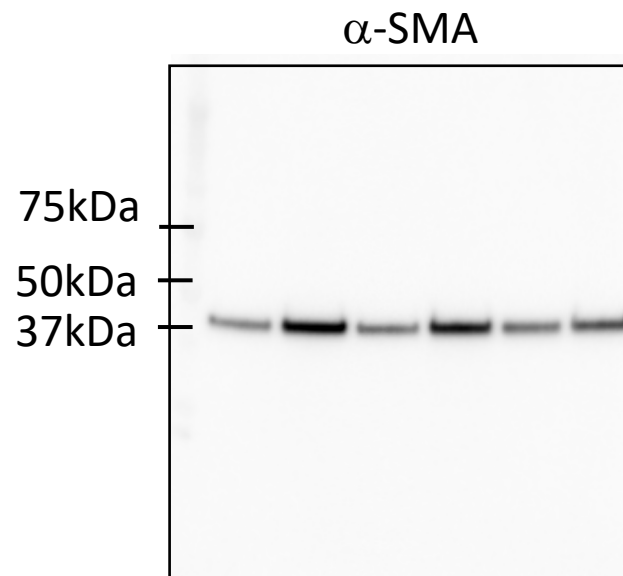

# Figure 4D

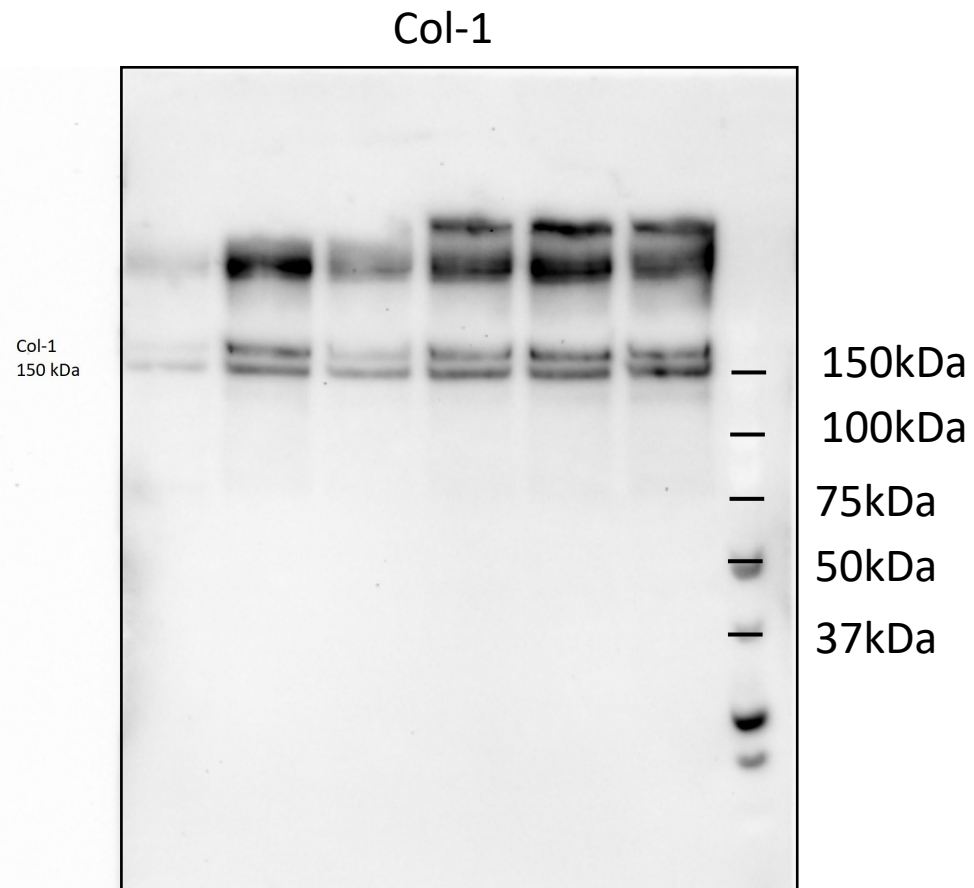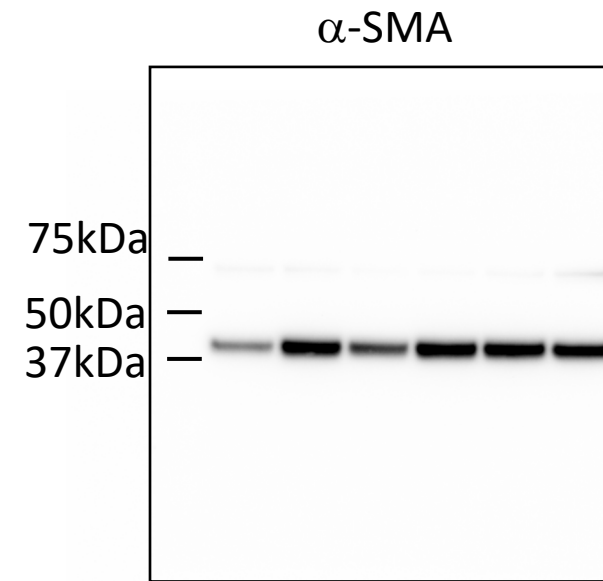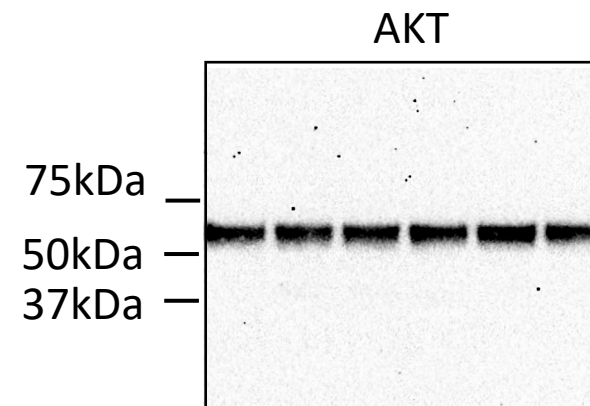

# Figure 4E

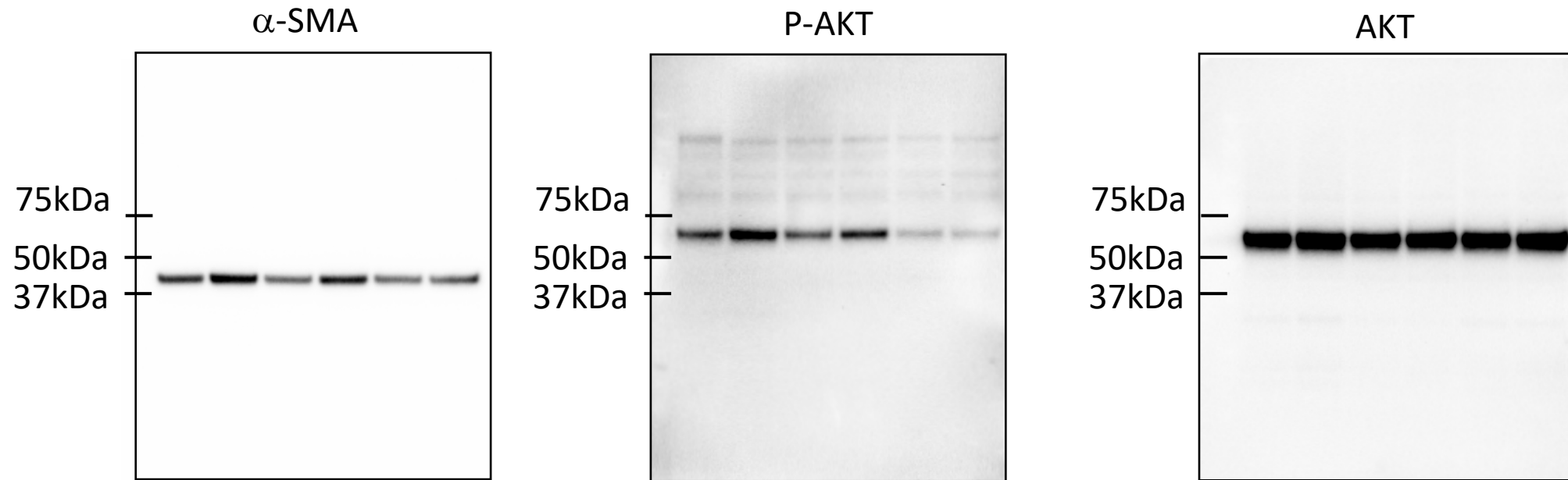

# Figure 5

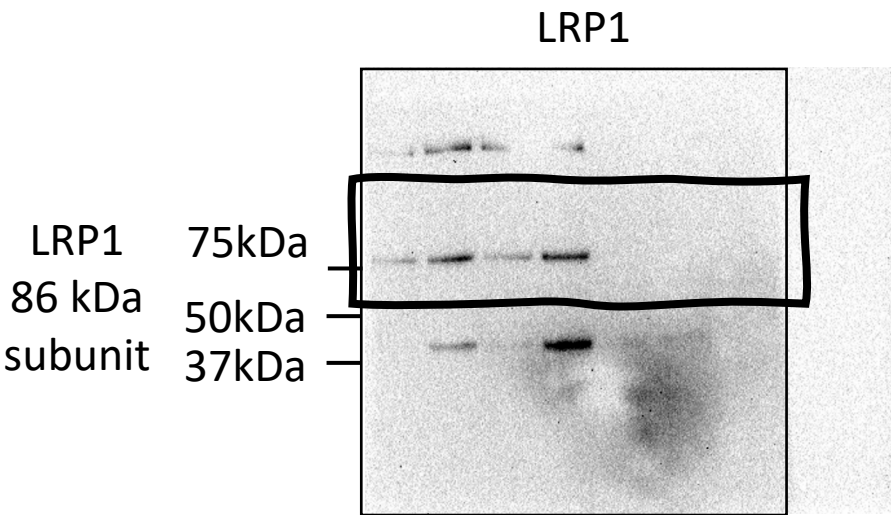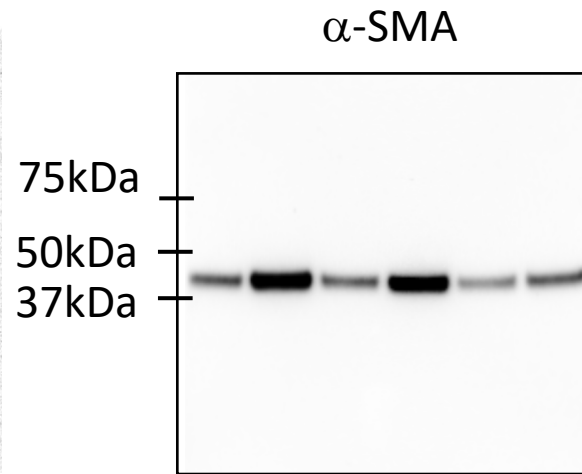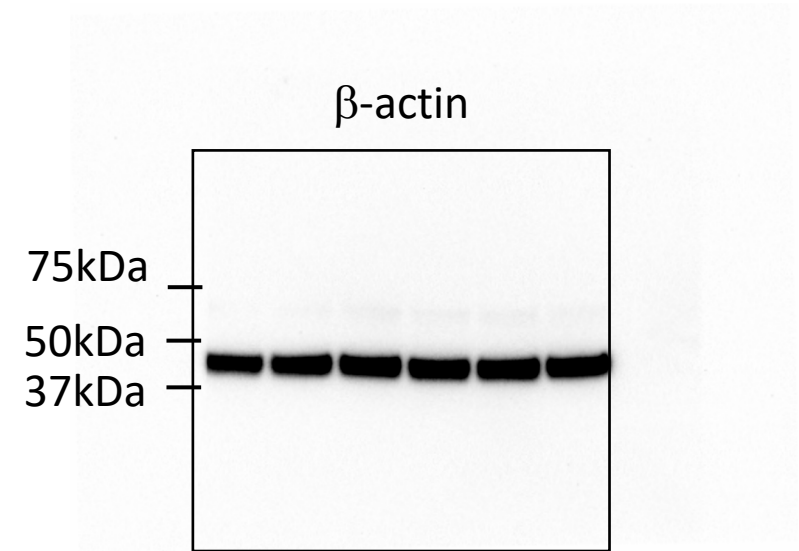

Supplement: Supplementary file 2 — Figure Deck for the manuscript. [file 41598_2021_99520_MOESM2_ESM.pdf]
